# Young White Pine Detection Using UAV Imagery and Deep Learning Object Detection Models

**DOI:** 10.3390/s26041284

**Published:** 2026-02-16

**Authors:** Abishek Poudel, Eddie Bevilacqua

**Affiliations:** Department of Sustainable Resources Management, State University of New York College of Environmental Science and Forestry, Syracuse, NY 13210, USA; ebevilacqua@esf.edu

**Keywords:** forest monitoring, regeneration, confusion matrix, F-RCNN

## Abstract

This study demonstrates the power of combining unmanned aerial vehicle (UAV) imagery and deep learning (DL) for monitoring forest regeneration, specifically focusing on young white pine (*Pinus strobus*). Using high-resolution three-band RGB and five-band multispectral orthomosaics derived from UAV flights, 20 DL object-detection models were evaluated within ArcGIS Pro 3.4 software (Esri Inc., Redlands, CA, USA). The models were tested across study sites in St. Lawrence County, NY, to assess performance on three distinct size classes of white pine, each stratified into low, medium, and high density areas. The Faster R-CNN (F-RCNN) model, particularly when trained with image rotation and no augmentation, significantly outperformed others, achieving an average precision of 0.88 across both imagery types. Subsequent confusion matrix analysis yielded 91% and 90% overall accuracy in medium and high-density white pine blocks, respectively. These findings validate the use of UAV-DL systems as an accurate and efficient tool for operational white pine regeneration assessment, reducing the need for labor-intensive fieldwork.

## 1. Introduction

Effective monitoring of young plantation forests and post-harvest regeneration areas is critical for sustainable forest management. It enables tracking reforestation outcomes, timber resources planning, and design of appropriate silvicultural interventions [1,2,3,4,5]. Traditional ground-based inventories, while accurate, are constrained by high costs, logistical complexity, and limited spatial coverage, hindering comprehensive assessment, particularly in large or inaccessible plantation areas [6,7,8].

Recent advances in unmanned aerial vehicle (UAV) technology, particularly platforms equipped with high-resolution digital cameras, primarily standard RGB sensors, offer a transformative solution for acquiring spatially detailed data. UAVs can collect imagery with a ground sampling distance (GSD) of 7 cm or less, sufficient to detect individual seedlings and juvenile trees [9]. Structure-from-Motion (SfM) photogrammetry applied to overlapping UAV images enables the generation of dense 3D point clouds and Canopy Height Models (CHMs), yielding valuable structural metrics such as tree height and crown area [5,10,11].

Historically, automated individual tree crown detection and delineation (ITCD) from high-resolution optical imagery relied on methods such as local maxima (LM) filtering of brightness or CHM surfaces, valley/region growing, watershed segmentation, template matching, and active contour models [12,13,14]. While applicable in certain forest types, these traditional algorithms often face limitations in young, dense plantations or regeneration areas due to factors like crown overlap, complex backgrounds with competing vegetation, sensitivity to parameter tuning, and difficulty distinguishing target seedlings from natural regeneration, particularly in hardwood stands.

The rapid development of deep learning (DL), especially Convolutional Neural Networks (CNNs), has revolutionized image analysis across many fields, including remote sensing and forestry [5,15,16]. CNNs excel at automatically learning hierarchical features directly from image data, overcoming many limitations of traditional handcrafted feature-based methods and often achieving State-of-the-Art performance in tasks like object detection and segmentation. The typical DL workflow involves data acquisition, primarily UAV RGB imagery for young forests, preprocessing, data augmentation, model selection, training, and evaluation [15].

Many studies have demonstrated the successful application of various CNN architectures for ITCD in young plantation forests and regeneration sites using UAV RGB data [9,17,18,19]. Common architecture includes two-stage detectors like Faster R-CNN [9,17,18] and Mask R-CNN [1,20,21], which perform instance segmentation (pixel-level delineation), and one-stage detectors like You Only Look Once (YOLO) [4,18,22] and Single Shot Detectors (SSD) [18,19], which prioritize speed. Other networks like RetinaNet, U-Net [23] have also been employed. These DL models consistently show high accuracy for detecting and counting seedlings [19,22], delineating individual crowns, measuring crown dimensions [18], and sometimes predicting tree height simultaneously [21], often outperforming classical methods like LM or watershed segmentation in comparative studies [24,25]. Furthermore, features extracted from DL-delineated crowns can be used with classifiers (e.g., Random Forest) for accurate species classification, with standard RGB imagery proving sufficient in some cases [26].

More recently, object detection research has advanced beyond standard CNN-based architectures toward approaches that improve efficiency, reduce annotation requirements, and better accommodate complex aerial scenes. Newer one-stage detectors, such as YOLOv11, have demonstrated improved performance for small and visually complex targets in UAV remote sensing imagery through enhanced feature representation and network design [27]. In parallel, weakly supervised object detection has gained traction in remote sensing, leveraging image-level supervision and multiple-instance learning to reduce dependence on exhaustive annotations while maintaining effective localization performance [28]. Additionally, recent review studies highlight growing interest in orientation-aware, context-aware, and transformer-based detection frameworks for individual tree crown detection, which aim to better represent irregular or overlapping objects in dense forest environments [5]. While promising, many of these emerging approaches remain less accessible within operational GIS-based workflows and are therefore discussed here to contextualize recent methodological trends rather than as methods evaluated in this study.

Despite significant progress, several challenges persist in applying DL for young forest monitoring. The dependency on large amounts of accurately annotated training data remains a primary obstacle. Strategies to address this include extensive data augmentation, transfer learning, and using synthetically generated training data [17], semi-supervised learning leveraging noisy labels from unsupervised methods [23], and developing fully unsupervised detection pipelines [29,30]. Model transferability and generalization across different sites, sensor types, species compositions, and environmental conditions remain critical, often requiring domain adaptation or site-specific fine-tuning.

To assess the generalization and transferability of DL models to different site conditions, this study aims to evaluate the ability of DL models to accurately identify and delineate individual trees across variable stand conditions. Specifically, this study investigates the effectiveness and generalizability of DL object-detection models for detecting regenerating white pine (*Pinus strobus*) in a plantation forest located within the Adirondack Park, New York, using high-resolution UAV imagery. To evaluate model robustness and generalization, the site was stratified into three blocks representing low-, medium-, and high-density regeneration based on stems/ha. The low-density (LD) block was used for model training, while the medium-density (MD) and high-density (HD) blocks were reserved for testing. This stratified approach enabled an evaluation of model transferability across differing regeneration densities.

The study also examined the effects of data augmentation strategies and input imagery types (RGB vs. multispectral) under rotated and non-rotated training conditions. A hybrid accuracy assessment framework was introduced, combining standard DL evaluation metrics with confusion matrix–based validation, including true negative (TN) estimations derived from “Other” class buffers for accuracy assessment. Finally, results were contextualized with findings from classical delineation methods, including watershed segmentation and LM filtering, to highlight differences between DL-based detection and traditional crown delineation workflows.

Although UAV-based DL methods for individual tree detection are increasingly common, most existing studies emphasize algorithm development or benchmark performance rather than their use within operational, GIS-based workflows relevant to forest management. As a result, how stand density, data augmentation strategies, and sensor type influence model performance and transferability under real-world management conditions remains insufficiently examined. This study evaluates the performance and generalizability of DL object-detection models available in ArcGIS Pro for detecting regenerating white pine from UAV imagery, with a focus on applied forestry use. Specifically, we compare multiple ArcGIS-supported detection architectures across varying regeneration densities, assess the effects of rotation-based data augmentation and input imagery type (RGB versus multispectral), and implement a hybrid accuracy assessment framework that integrates standard deep-learning metrics with confusion-matrix-based validation to support operational forest regeneration monitoring.

## 2. Materials and Methods

### 2.1. Study Area

**Figure 1:**
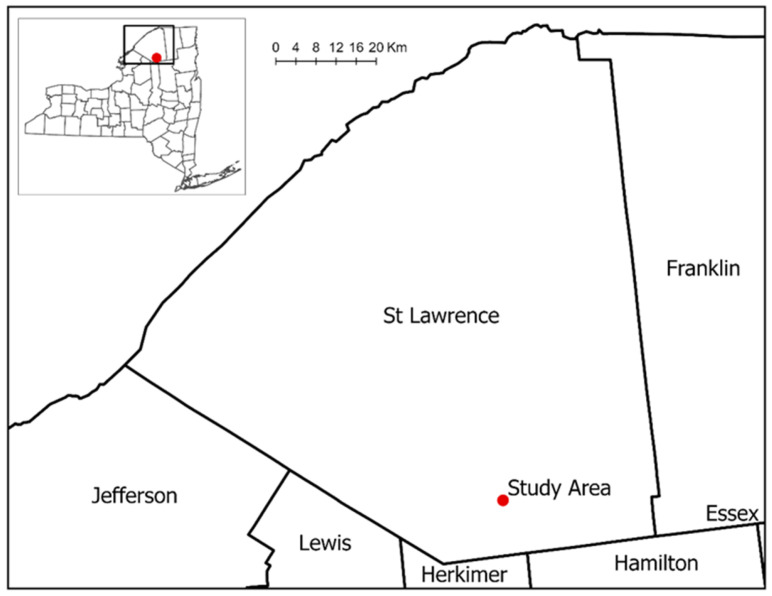
Map showing the location of the study area in Adirondack Park, New York.

**Figure 2:**
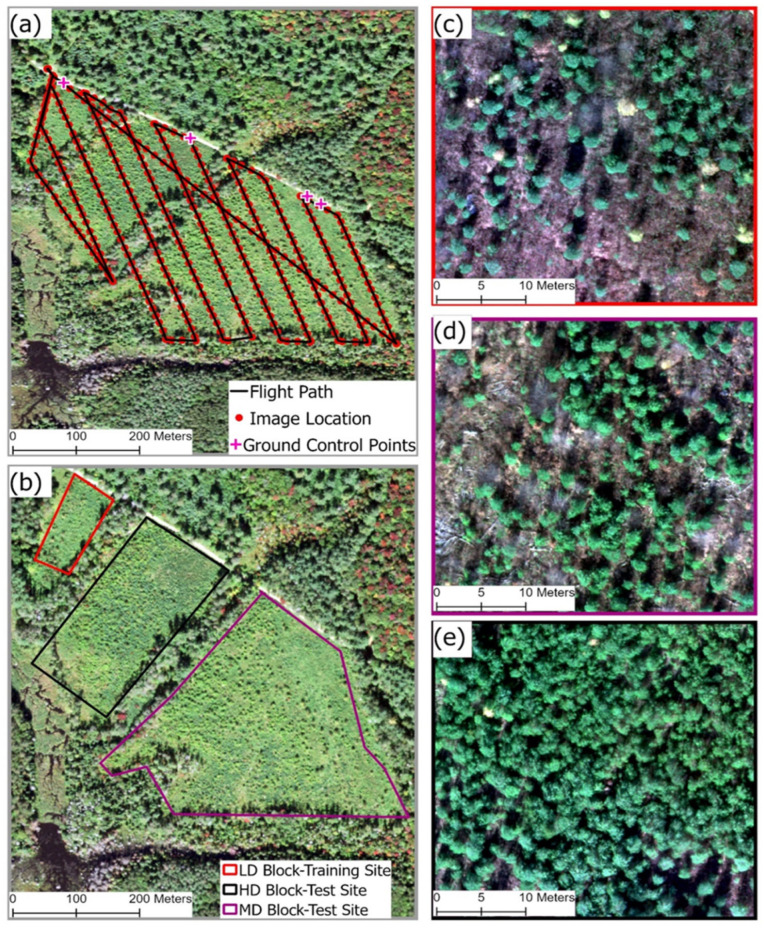
(**a**) The study area map illustrating a single grid UAV flight pattern, ground control points (GCPs), and image location depicted with red dots. (**b**) Location of training and testing site; (**c**–**e**) are examples of UAV-derived multispectral imagery with 6 cm spatial resolution for Low-Density (LD) Training Site (**c**), High-Density (HD) Test Site (**d**), and Medium-Density (MD) Test Site (**e**).

The study was conducted within a managed white pine (*Pinus strobus* L.) plantation block located in the Adirondack Park, New York (Figure 1), under the management of the State University of New York (SUNY) College of Environmental Science and Forestry (ESF). Following a complete harvest in 2013, the three distinct experimental blocks within the study site were replanted with white pine and, for this study, named as low, medium, and high density based on their respective white pine stocking (Figure 2b). The LD block encompasses an area of 0.65 hectares (ha) (1.6 acres), while the medium-density MD block covers 5.38 ha (13.3 acres); both were planted with white pine between 2014 and 2017. The HD block, covering 1.62 ha (4 acres), was planted in 2015.

### 2.2. Field-Measurements

Field data collection was conducted in a random-systematic grid of fixed-area circular plots. Specifically, twenty-nine 1/500th-acre plots were established in the HD block, while thirty 1/200th-acre plots were established in the MD block and seven in the LD block. This differential plot-size allocation was implemented due to logistical constraints related to the number of field crew members and substantial regeneration within the HD site, necessitating smaller plot sizes for efficient data acquisition. The random-systematic sampling design involved establishing a random starting point and a transect line. To ensure that each plot included a planted tree, the nearest tree within one chain (66 feet, ~20 m) of the plot was selected as the plot center. This tree was then tagged using hanging labels marked with the corresponding transect line and plot number, allowing for accurate identification and spatial referencing. Transects were navigated using compass bearings of 220° (southwest) and tape measures, with plots spaced at one chain (66 feet, ~20 m) intervals along each transect. Within each established plot, all young regenerations above 0.3 m in height were tallied. For each tallied plant, collected data included species identification, diameter at the root collar (mm), total height (cm), and average crown width (cm). Additionally, the distance and azimuth of each plant from the plot center were measured to determine spatial distribution patterns. Each plot-centered tree was tagged with a unique line and plot number. The precise geographic coordinates of each plot center were recorded using a Trimble Geo XH 3000 global positioning system (GPS) unit (Trimble Inc., Westminster, CO, USA). GPS data were post-processed using Trimble GPS Pathfinder Office 5.81 software (Trimble Inc., Westminster, CO, USA). which applied differential correction. This method used data from established base stations to correct positional errors in field-collected data, achieving sub-meter accuracy within a range of 10 to 50 cm.

Plot-level summary statistics were calculated for tree count, average crown width, and total height to provide an overview of white pine characteristics within each density block. These statistics, including the mean, median, standard deviation, minimum, and maximum values, are presented in Table 1 for the Low Density (LD), Medium Density (MD), and High Density (HD) blocks. Stem density (stems/ha) for each block was calculated by scaling the average plot-level stem count by its corresponding plot expansion factor. The initial estimate in stems per acre was then converted to stems per hectare using a conversion factor of approximately 2.471. Plot expansion factors of 500, 200, and 200 were used for the HD, MD, and LD blocks, respectively. This resulted in estimated densities of 8796 stems/ha in the HD block, 4744 stems/ha in the MD block, and 3657 stems/ha in the LD block. These initial summary statistics provide a foundational understanding of the structural characteristics of the white pine within the different density blocks before further analysis.

### 2.3. Unmanned Aerial Vehicle Data Collection and Processing

Aerial imagery acquisition for this study was conducted in November 2021, utilizing a DJI Matrice 100 Unmanned Aerial Vehicle (UAV) (DJI, Shenzhen, China) equipped with a nadir-facing Micasense RedEdge-M multispectral camera (mounted below the aircraft batteries). Flight planning, including determining flight speed, camera gimbal angle, and flight altitude, was performed using Pix4Dcapture software (Pix4D SA, Lausanne, Switzerland). To ensure accurate georeferencing of the imagery, four Ground Control Points (GCPs) were established. While a dispersed layout is often ideal, the GCPs were placed strategically in a linear pattern along the road. This was a necessary adaptation to field conditions, ensuring the GCP’s target would be visible in the final imagery and not obscured by the site’s vegetation and ground cover. The precise geographic coordinates of each GCP were recorded using a Trimble Geo XH 3000 GPS unit (Trimble Inc., Westminster, CO, USA). The UAV flight was conducted under clear atmospheric conditions. A flight altitude of 93 m was specifically chosen to safely clear the mature trees surrounding the study area. A single grid flight pattern with a high image overlap (front and side) of 80% was used to ensure complete site coverage and provide the robust data required for generating an accurate 3D model and detecting regenerating white pine (Figure 2a). The flight planning acquisition parameters are detailed in Table 2.

The UAV imagery was processed using Pix4Dmapper 4.8.4 (Pix4D SA, Lausanne, Switzerland). During initial processing, the software successfully calibrated all 1740 images. This means it determined the precise position and orientation for each photo by finding a high number of matching points between them (a median of 7537 matches per image). This robust reconstruction resulted in a final Ground Sample Distance (GSD) of 6.32 cm. GCPs’ data was imported into Pix4Dmapper after initial processing for georeferencing UAV-derived images. The resulting RMSE for the GCPs was 0.23m. Advanced image processing generated various data products (Table 3), including reflectance indices for the five spectral bands (Blue, Green, Red, NIR, and Red Edge). The image processing was completed within 2 h and 13 min.

### 2.4. Training Datasets Generation

**Figure 3:**
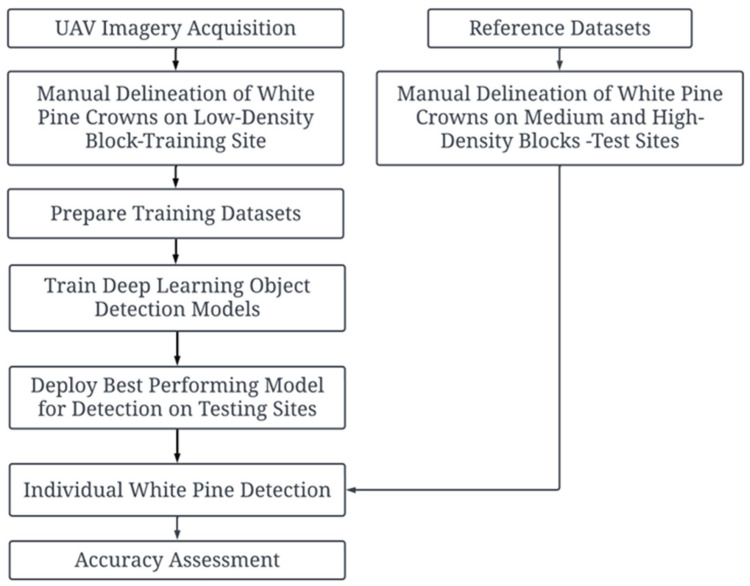
A workflow for detecting regenerating white pines in the study.

The overall framework of this study, from data acquisition to accuracy assessment, is presented in Figure 3. The proposed automated individual white pine regeneration detection methodology relies on implementing the best-performing object-detection model identified from a suite of models available within the ArcGIS Deep Learning Libraries (ArcGIS Pro 3.4). This selection process involved training and evaluating several DL architectures on the manually delineated training dataset derived from the LD test site. The model with the highest performance metrics based on average precision on a validation subset of the training data was selected as the basis for automated regeneration detection at the independent MD and HD block test sites.

**Figure 4:**
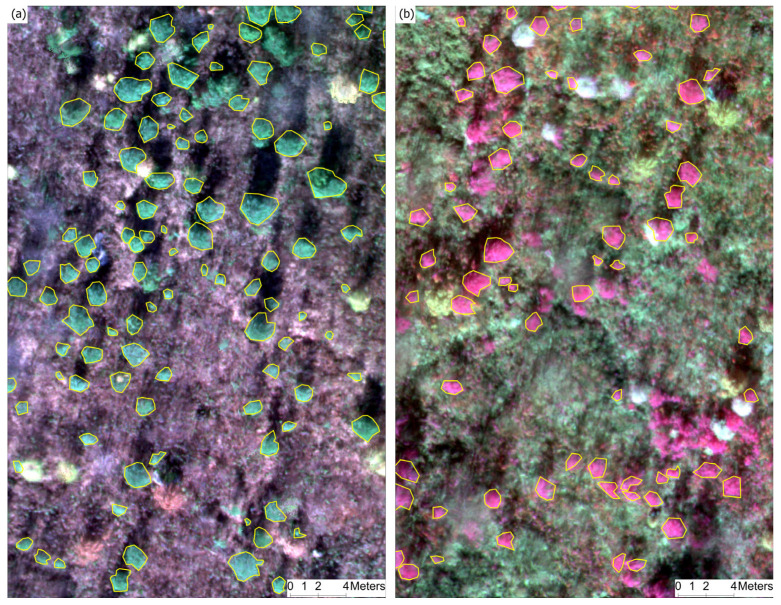
(**a**) Manually delineated white pine crowns from the LD block using natural color imagery (**Left**) and false color imagery (**b**) in which vegetation is displayed in pink due to near-infrared reflectance (**Right**).

**Figure 5:**
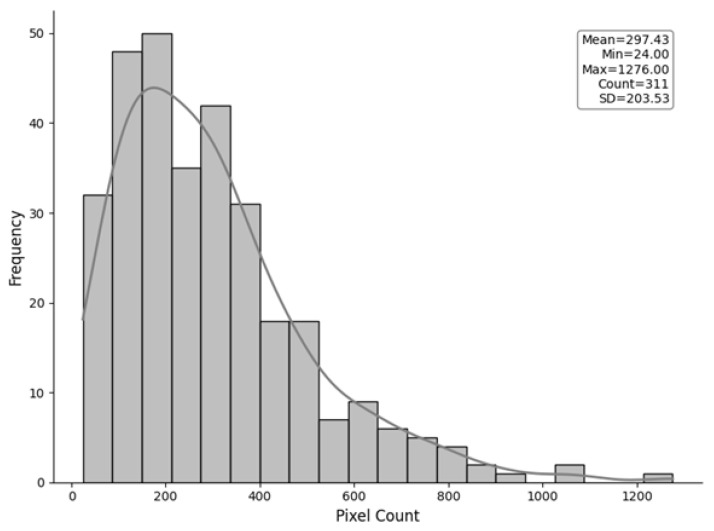
Histogram showing the distribution of training samples created for the purpose of training data generation.

Creating training data for DL object-detection models involved a meticulous manual delineation process within ArcGIS Pro 3.4. High-resolution multispectral orthomosaic imagery of the LD study site served as the primary data source. Band-combination techniques were applied to generate natural-color and false-color composite imagery from multispectral imagery and to accurately identify individual regenerating white pines. The false-color composite was created by displaying the Near-Infrared (NIR) band in the red channel. Since healthy vegetation strongly reflects NIR light, this technique creates a high-contrast image in which the crowns of white pine appear in a distinct, bright color (often red or magenta), separating them from the soil, dormant vegetation, and other background features. This enhanced visual separation between the trees and the surrounding background facilitates the precise and accurate identification of individual trees. Using ArcGIS Pro’s training sample manager, the interpreter iteratively identified and digitized the boundaries of each discernible white pine crown based on natural color (Figure 4a) and false color (Figure 4b) imagery, resulting in a comprehensive training dataset of labeled polygons spatially defining individual white pine regeneration within the LD block study site (Figure 4). Figure 5 illustrates the distribution of pixel counts within these manually delineated crowns, ranging from 24 to 1276 pixels, with a mean of 297.43 and a standard deviation of 203.53 (*n* = 311). This distribution highlights the natural variability in the size and spatial extent of the white pine regeneration within the LD training site.

Training datasets were generated using three-band RGB and multispectral imagery. The ArcGIS Pro Image Analyst tool, “Export Training Data for Deep Learning”, was employed to convert the labeled white pine crown polygons into DL training data, leveraging the RGB and multispectral imagery of the study area (Table 4). This process resulted in an output folder containing image chips and associated metadata files, formatted according to ArcGIS Pro’s default PASCAL Visual Object Classes (VOC) standard for object detection. The PASCAL VOC format structures the output as a collection of image chips, each accompanied by an XML file. These XML files detail the bounding box coordinates and the corresponding labels for the white pine objects present within each image chip. This standardized output is then directly suitable for training DL object-detection models within the ArcGIS Pro environment. For multispectral training and inference, the orthomosaic was used as a five-band raster (Blue, Green, Red, Red Edge, and NIR) and exported as five-channel image chips; ArcGIS Pro handled the multi-band stacking internally during chip export, model training, and inference.

Increasing the size of the training dataset is standard practice and beneficial for DL-based object detection, particularly in complex applications like tree detection from remote sensing imagery. This is primarily because it enhances model accuracy and generalization while mitigating overfitting. However, the benefits of adding more data are subject to diminishing returns, when adding more data yields progressively smaller gains in accuracy [31].

In this study, four distinct training datasets were created with RGB and multispectral imagery, processed with 0° and 45° rotation angles to expand the data. RGB and multispectral imagery were divided into tiles of 256 × 256 pixels for training sample extraction. A stride of 128 × 128 pixels was used, meaning the sliding window that extracts each tile moved 128 pixels horizontally and vertically between successive tiles. This configuration results in overlapping tiles, increasing the likelihood of capturing complete objects, particularly those near tile boundaries, and improving training data coverage. This approach aligns with standard practices, where data augmentation techniques, such as geometric transformations like rotation, are used to artificially expand datasets [22]. By presenting objects from multiple angles, these techniques force the model to learn orientation-independent features, making it more robust. This increased data diversity helps reduce the risk of overfitting and enables more effective generalization across diverse and challenging environments [32].

**Table 4:** Parameters used in creating training data in ArcGIS Pro to train the DL object-detection model [33].

Items	Input	Description
Imagery	RGB, Multispectral	Imagery used to create training data.
Training Sample	White Pine’s Crown Polygons	Training samples created by the interpreter.
Image Format	TIFF	Raster format for the image chip outputs.
Tile Size X	256	Image chip’s size in the x-dimension.
Tile Size Y	256	Image chip’s size in the y-dimension.
Stride X	128	x-direction shift for image chip creation.
Stride Y	128	y-direction shift for image chip creation.
Metadata Format	PASCAL VOC	Metadata format of image chips.
Rotation Angle	0°, 45°	Rotation angle used to generate image chips.

### 2.5. Deep Learning Object Detection Architectures

Object detection is a key task in computer vision, focusing on identifying and locating objects within images, and involves predicting bounding boxes and corresponding class labels [34]. DL has driven significant progress, with models often distinguished by their reliance on predefined anchor boxes and whether they perform detection in one or two stages. A fundamental distinction lies between anchor-based models, which use predefined reference boxes (anchors) to guide localization, and anchor-free models, which predict objects directly from image features.

Anchor-based object detectors include two-stage models like Faster R-CNN, which introduced the Region Proposal Network (RPN) [35]. Enhancements followed with Libra R-CNN, focusing on balanced training [36], and Cascade R-CNN, which improved localization via multi-stage refinement [37]. For greater efficiency, single-stage detectors like SSD and YOLOv3 [38] perform detection in one pass, while RetinaNet introduced focal loss to address class imbalance [34].

Anchor-free detectors emerged to reduce anchor complexity. Fully Convolutional One Stage (FCOS) predicts bounding box distances from feature points using a center-ness score [39], while FoveaBox [40] and Vari Focal Net (VFNet) [41] further improve detection via intersection over union (IoU)-aware scoring and Vari Focal loss. Hybrid methods bridge these paradigms, such as Feature Selective Anchor Free (FSAF), which adds anchor-free branches to anchor-based models [42] and ATSS, which introduces adaptive training sample selection [43].

Detection accuracy also benefits from enhanced feature representations, such as Feature Pyramid Networks (FPN), Path Aggregation FPN (PaFPN) for improved flow [44], and Res2Net for fine-grained multi-scale features [45]. Modules such as Deformable Convolution Network (DCN) [46], Content Aware Reassembly of Features (CARAFE) for upsampling [47], GHM for gradient balance [48], Cascade RPN [49], and Side Aware Boundary Localization (SABL) for bounding box localization [50] further support performance.

Overall, object detection is trending towards simplified, efficient pipelines (anchor-free, one-stage), better feature encoding (FPN, DCN), improved localization (VFNet, SABL), and robust training (Focal Loss, ATSS, Libra R-CNN), with modular designs enabling cross-framework innovation.

This study examined the performance of DL models, influential components, and techniques as discussed above and available within ArcGIS Deep Learning Libraries, specifically those compatible with the PASCAL VOC metadata. The ArcGIS deep learning framework is implemented through the arcgis.learn Python API and is built upon widely adopted open-source libraries, including PyTorch and fastai [51]. As a result, models evaluated in this study, including Faster R-CNN with a ResNet backbone, follow the same underlying architectures and learning formulations as their standalone counterparts. Accordingly, the focus of this work is on evaluating operational, GIS-integrated DL workflows rather than on developing or benchmarking custom models. The DL models examined in this study are listed in Table 5, along with their abbreviations when available.

### 2.6. Model Training and Application

The Automated Deep Learning (AutoDL) tool within ArcGIS Pro 3.4 established an initial baseline for object detection performance. The ‘Advanced AutoDL Mode’ was utilized to evaluate the performance of multiple standard DL architectures. The process involved screening a broad set of architectures against our training data using a default feature extractor (backbone). It then took the top two performing architectures and tested them thoroughly with a variety of alternate backbones. This process automates data augmentation, optimal model architecture selection, hyperparameter optimization, and batch size determination. The evaluation included the object detection architecture available in ‘AutoDL’, which is listed in Table 5. Model performance was evaluated using the average precision metric.

The top-performing models are listed in Table 6, along with average precision scores for models trained with RGB and multispectral imagery with and without image rotation.

Based on initial screening and subsequent evaluations conducted across various scenarios, encompassing various imagery types and considerations for image rotation, the top three architectures that exhibited notable consistency and superior performance were identified (Table 6). These models are F-RCNN, FoveaBox, and PaFPN. Consequently, these three models were selected for further in-depth analysis in the study.

Training DL models for object detection with “Train Deep Learning Model” in ArcGIS Pro involves selecting architectures representing different strategies. Among the high-performing models selected were Faster R-CNN (F-RCNN), an anchor-based, two-stage framework; FoveaBox, an anchor-free, one-stage method; and PaFPN, a feature enhancement module. F-RCNN operates as a landmark two-stage framework employing RPN that shares features with the detection network, significantly improving efficiency over predecessors by integrating proposal generation [35]. FoveaBox is an anchor-free, one-stage object detection method [40,52] that directly predicts object properties from central feature locations, positioned as moving “Beyond Anchor-Based Object Detection” with specific mechanisms detailed in its primary source [40]. PaFPN enhances FPN by adding a bottom-up path augmentation and using adaptive feature pooling [44], improving multi-scale feature flow for better detection, and typically functioning as the feature fusion neck. ArcGIS Pro manages the configuration and training of these architectures according to the user’s selections, with the corresponding parameters and values presented in Table 7. To evaluate the impact of data augmentation, models were trained under two separate conditions. The first model was trained using the default augmentation pipeline, which dynamically applies random transformations such as zoom, crop, contrast, and brightness to the training data, creating new, slightly altered training samples. For comparison, the second model was trained with all on-the-fly augmentations disabled.

The selected DL architectures—F-RCNN, FoveaBox, and models incorporating PaFPN—were trained using the “Train Deep Learning Model” tool in ArcGIS Pro to detect white pine regeneration. A stratified subset comprising 10% of the labeled data was reserved as a validation set to monitor model performance during training. ArcGIS Pro manages the training workflow, enabling the models to learn to localize potential white pine regeneration through bounding box prediction.

Model performance was assessed using the IoU metric, which quantifies the spatial overlap between predicted and ground reference bounding boxes. Following ArcGIS Pro’s default evaluation protocol, a threshold of 0.5 was used for training-stage IoU-based validation, while all reported detections and accuracy assessments in this study were filtered using a confidence threshold of 0.95. The IoU-based validation is a critical component of ArcGIS Pro’s internal validation process and contributes to the calculation of standard performance metrics, including precision and recall.

### 2.7. Testing with Independent Sites

Following the completion of the training phase, white pine regeneration within the UAV-derived orthomosaics was detected utilizing the “Detect Objects Using Deep Learning” tool in ArcGIS Pro. To evaluate the generalization and transferability of the model, a top-performing model trained on data from the LD block test site was applied to orthomosaics corresponding to the MD and HD block test sites. Furthermore, the highest-performing architecture was selected for further analysis to assess the impact of data augmentation strategies.

Achieving optimal detection performance requires meticulous configuration of the detection tool parameters, the specific values of which are detailed in Table 8. Padding was established at 56 pixels in all four directions of the image tile to ensure consistent prediction across image tile boundaries. An inference batch size of 32 dictated the concurrent processing of image tiles. Detections with confidence scores below the threshold of 0.95 were excluded from consideration, and non-maximum suppression (NMS) was employed to address redundancy by retaining only the white pine regeneration detection with the highest confidence score among overlapping bounding boxes. This detection procedure resulted in a feature layer that included predicted bounding boxes and their respective confidence scores for the identified white pine regeneration at the MD and HD study sites. All computational tasks were performed on a high-performance computing system with a Windows 11 Enterprise operating system, an Intel Xeon Silver 4214 CPU (Intel Corporation, Santa Clara, CA, USA), an NVIDIA RTX A6000 GPU (NVIDIA Corporation, Santa Clara, CA, USA), and 64 GB of RAM. 

### 2.8. Reference Dataset

**Figure 6:**
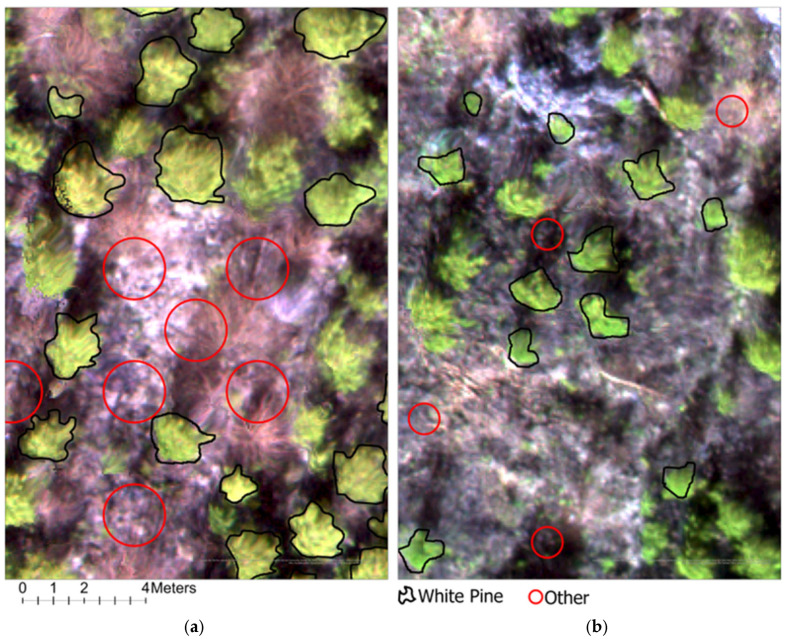
Manually delineated polygons of white pine and other trees in (**a**) the HD block (**Left**) and (**b**) the MD block (**Right**).

A detailed ground reference dataset was manually developed in ArcGIS Pro 3.4 for object detection accuracy assessment. This process was based on high-resolution multispectral imagery acquired from the MD and HD blocks. The visible white pine crowns were manually digitized using ArcGIS Pro’s training sample manager. For accuracy assessment, 500 white pine reference polygons were independently delineated for each of the MD and HD blocks to represent target regeneration crowns. These reference polygons were distributed across each block to capture spatial variability in regeneration density and canopy conditions (Figure 6).

The TN data representing the “Other” class were also systematically generated to enable a complete confusion matrix-based accuracy assessment. It involved overlaying a 1 m × 1 m grid on each testing block and developing a point feature at the center of each grid cell. These points were then buffered based on study site-specific average crown width values—0.5 m for the MD block and 1 m for the HD block. The buffered polygons were carefully filtered to remove any that intersected with the manually delineated white pine crown polygons. The remaining polygons, representing open space areas, were labeled as “Other” (Figure 6). This yielded 500 “Other” polygons for the MD block and 400 for the HD block.

### 2.9. Accuracy Assessment

Accuracy assessment was based on the evaluation of true positives (TP), false positives (FP), false negatives (FN), and true negatives (TN) using a polygon-level, map-based confusion matrix constructed from model detections and reference data. Reference data consisted of polygons labeled as “White Pine” (target class) and “Other” (non-target class). Detection outcomes were evaluated as follows:

- True Positive (TP): A reference polygon labeled “White Pine” that intersects at least one predicted bounding box, indicating a correctly detected white pine crown.
- False Negative (FN): A reference polygon labeled “White Pine” that does not intersect with any predicted bounding box, representing a missed white pine crown.
- False Positive (FP): A predicted bounding box that intersects a reference polygon labeled “Other”, indicating a commission error into non-target vegetation.
- True Negative (TN): A reference polygon labeled “Other” that does not intersect with any predicted bounding box, representing correct non-detection of non-target areas.

Based on TP, FP, FN, and TN, precision, recall, and overall accuracy (OA) were derived:

(1) $$Precision=\frac{TP}{TP+FP}$$

(2) $$Recall=\frac{TP}{TP+FN}$$

(3) $$OA=\frac{TP+TN}{TP+FP+FN+TN}$$

These metrics represent polygon-level accuracy and are not intended to replicate classical object-detection evaluations based solely on bounding-box IoU matching. While ArcGIS AutoDL internally reports standard object-detection metrics using bounding-box overlap, the accuracy assessment presented here was conducted using polygon-level confusion matrices constructed from the spatial intersection of model detections and independently delineated reference polygons for both the MD and HD blocks. The assessment followed a binary evaluation framework distinguishing “White Pine” (target) and “Other” (non-target) reference classes, as described in Section 2.8.

## 3. Results

### 3.1. Model Performance

**Figure 7:**
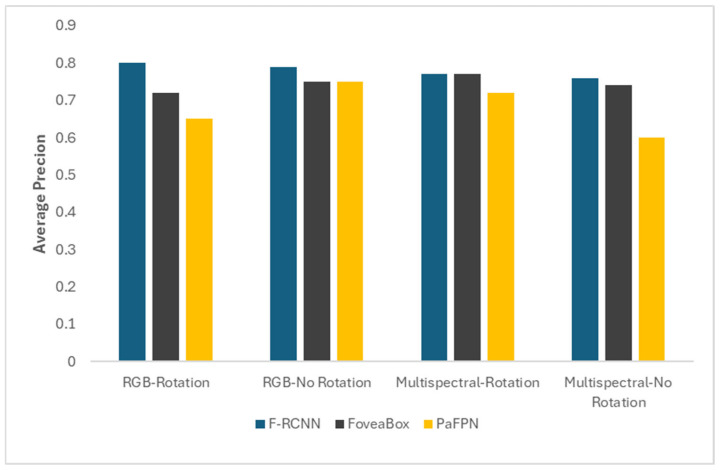
Average precision score of the top three DL object-detection models tested on RGB and multispectral imagery, comparing results with and without image rotation.

The initial evaluation of various DL architectures using the ‘Advanced AutoDL Mode’ revealed significant variability in performance across models and tested conditions (Table 6). Notably, the F-RCNN architecture consistently achieved the highest average precision scores across all four scenarios, with its peak performance of 0.80 average precision occurring with three image bands and image rotation (45°), as illustrated in Table 6 and Figure 7. FoveaBox also demonstrated strong performance, although its average precision experienced a noticeable decline with multispectral bands and image rotation (0.60 average precision). PaFPN exhibited mid-range performance, showing variability depending on the scenario, and was among the better performers compared to many other architectures evaluated (GHM, Cascade RPN, FSAF, DynamicRCNN, SingleShot, YOLOv3).

### 3.2. Data Augmentation

**Figure 8:**
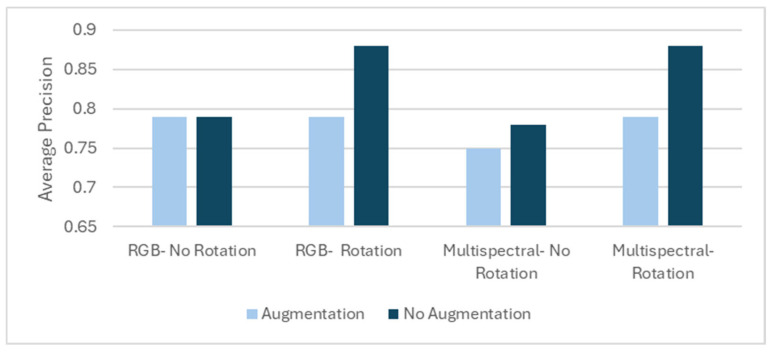
Average precision score for the F-RCNN model, tested on RGB and multispectral imagery, with and without rotation, comparing results with and without data augmentation.

Based on the AutoDL screening results, Faster R-CNN was selected for subsequent analysis due to its consistently higher average precision across all tested scenarios (Figure 8). Performance differences between input configurations were density dependent: a model trained with rotation and without augmentation using RGB imagery produced the strongest results in the MD block, whereas a model trained with rotation and without augmentation using multispectral imagery performed best in the HD block, indicating improved discrimination under dense regeneration conditions. Rotation-based data expansion increased the number of training image chips from 107 to 872. All Faster R-CNN models used a ResNet-50 backbone, with image chip size, stride, and overlap defined during the training dataset generation phase (Table 4). Multispectral inputs were processed as a single stacked raster containing all spectral bands, while RGB inputs were derived as three-band composites extracted from the same stack. Detailed training parameters are provided in Table 7.

The results revealed a notable and counterintuitive pattern: training the F-RCNN model without data augmentation consistently produced the highest or equivalent average precision scores in three of the four scenarios. In the RGB and multispectral with rotation conditions, the models trained without data augmentation achieved significantly higher average precision scores (0.88 in both cases) than the default augmentation (0.79 in both cases). A smaller performance gain was observed in the multispectral without rotation scenario, where the average precision increased to 0.78 without data augmentation, compared to 0.75 under default data augmentation. Both settings yielded identical average precision scores in the RGB without rotation scenario (0.79).

These findings indicate that the default data augmentation strategy offered by ArcGIS Pro did not enhance model performance for this specific task and dataset. Augmentation appeared to hinder performance in rotation-involved scenarios, possibly due to the introduction of geometric transformations that did not align well with the spatial characteristics of regenerating white pine. Although data augmentation is commonly applied to improve model generalization, the results from this study demonstrate that omitting augmentation leads to superior performance, as measured by average precision.

### 3.3. White Pine Detection

**Figure 9:**
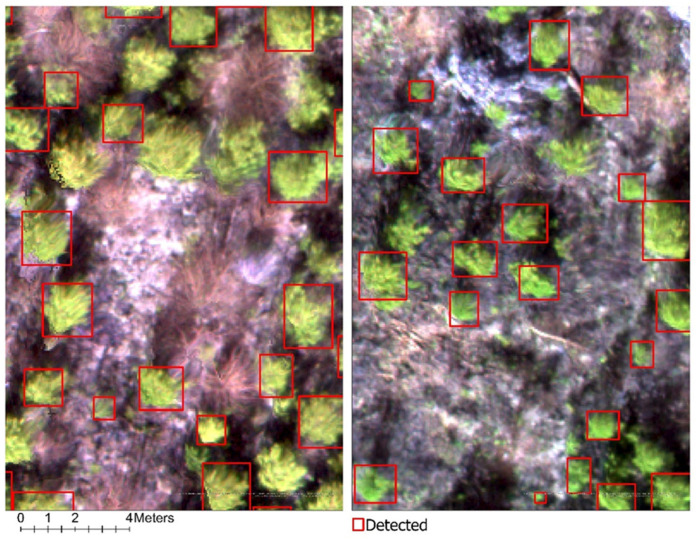
The map illustrates white pine detection with the F-RCNN object-detection model in the HD block (**Left**) and the MD block (**Right**). The detection shown was filtered using a high confidence threshold of 0.95.

Initial training and validation results identified F-RCNN as the most effective architecture for detecting regenerating white pine, outperforming alternative models. Detection was carried out on both the MD and HD blocks to examine model performance further using four input scenarios: RGB and multispectral imagery, each evaluated with and without image rotation. Two training configurations were compared—ArcGIS Pro’s default data augmentation and a no-augmentation baseline. All detections were filtered using a single, conservative confidence threshold of 0.95, such that only predictions with at least 95% confidence were retained for mapping, summary analysis, and accuracy assessment. This threshold was selected to emphasize high-certainty detections and to minimize false positives in complex forest backgrounds. The detection output consisted of bounding-box polygons delineating the predicted locations of individual white pine crowns (Figure 9).

**Figure 10:**
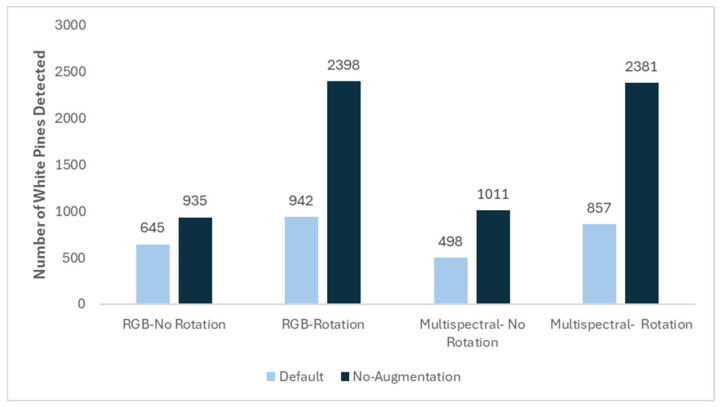
Number of white pines detected on the MD block using the F-RCNN model with a 0.95 detection threshold for RGB and multispectral imagery, with and without image rotation, and with and without data augmentation.

In the MD block, the model without data augmentation consistently outperformed the default augmentation model across all detection scenarios. Under the RGB with rotation condition, the model without data augmentation detected 2398 white pines, compared to 942 using default augmentation (Figure 10). Similarly, in the multispectral and rotation scenario, detections increased to 2381 without data augmentation, compared with 857 with augmentation. The non-rotated conditions also improved performance in the without-augmentation setting, with 935 detections in the RGB scenario compared to 645 with augmentation, and 1011 versus 498 in the multispectral scenario.

**Figure 11:**
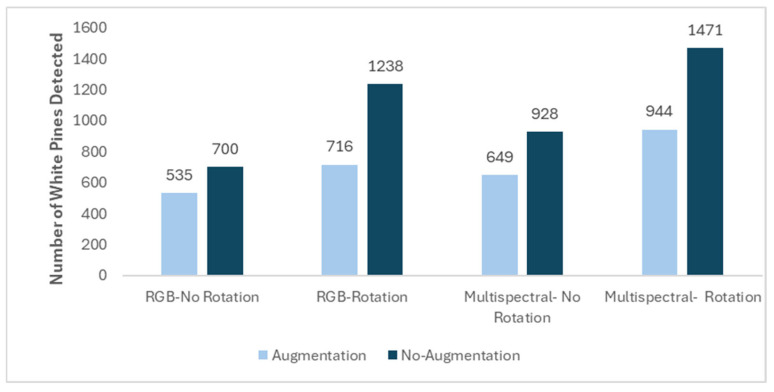
Number of white pines detected on HD block using F-RCNN model with 0.95 detection threshold for RGB and multispectral imagery with and without image rotation and with and without data augmentation.

Detection patterns in the HD block followed the same trend. In the RGB with rotation condition, the model without data augmentation detected 1238 white pines, substantially more than the 716 detected under default augmentation (Figure 11). A similar outcome was observed in the multispectral with rotation scenario, where 1471 trees were detected without augmentation, compared to 944 with augmentation. In non-rotated scenarios, the model performed better without augmentation, with 700 detections in the RGB condition versus 535 with augmentation, and 928 versus 649 in the multispectral condition.

These consistent gains across all scenarios suggest that the default augmentation strategy, particularly involving geometric transformations such as rotation, may have introduced variations misaligned with white pine regeneration’s spatial structure and spectral characteristics. Although data augmentation is generally applied to improve generalization, models trained without rotation-based augmentation achieved higher precision when only very high-confidence detections (confidence ≥ 0.95) were retained. This conservative confidence threshold was selected to reduce overlapping detections and visually evident FP that were frequently observed at lower confidence levels during visual verification.

### 3.4. Object Detection Performance Evaluation 

In this study, the detection performance of the F-RCNN model was evaluated using the independent reference polygons described in Section 2.8. For each block, 500 reference polygons labeled as “White Pine” were used to quantify omission (FN) and detection of target crowns (TP). To quantify commission into non-target areas, the “Other” reference polygons (500 in the MD block and 400 in the HD block) were used to compute FP and TN. All detections produced by the selected model configurations were considered, and the confusion-matrix results reported below were computed after applying a conservative confidence filter (confidence ≥ 0.95) to reduce low-confidence commission errors; the same threshold is used for the detection-count summaries in Figure 10 and Figure 11.

#### 3.4.1. Medium Density Block

The confusion matrix for the MD site shows that out of 500 reference "White Pine" polygons, 412 were correctly identified (TP) and 88 did not intersect any predicted bounding box (FN), representing missed crowns. Among the 500 “Other” reference polygons, only two were intersected by model detections, representing commission errors (FP), while 498 were correctly identified as non-target (TN). Based on these results, precision (0.99), recall (0.82), and OA (0.91) were derived for the F-RCNN model (Table 9).

These results indicate high precision and overall accuracy, with relatively few false positives. Recall is comparatively lower due to missed white pine crowns, indicating that while detections are highly reliable, some regenerating crowns remain undetected in the MD site.

#### 3.4.2. High Density Block

For the HD-block, the confusion matrix indicates that out of 500 reference "White Pine" polygons, 434 were correctly detected by the FRCNN model (TP), while 66 were not intersected by any predicted bounding box, representing missed crowns (FN). Among 400 reference polygons labeled as “Other”, 24 were intersected by model detections, representing commission errors (FP), while 376 were correctly identified as non-target areas (TN) (Table 10).

The model maintained high performance in the HD site as well, with strong precision (0.95), recall (0.87), and OA (0.90). Similar to the MD site, high precision reflects limited commission into non-target vegetation, while reduced recall indicates missed white pine crowns under dense regeneration conditions. The consistency in overall accuracy and error structure between the HD and MD sites suggests stable model performance across varying regeneration densities, although additional validation across a broader range of site conditions would be required to confirm broader transferability.

## 4. Discussion

### 4.1. Evaluation of Deep Learning Architectures

This study systematically evaluated multiple DL architectures within the ArcGIS Pro DL framework for detecting regenerating white pine in UAV-acquired RGB and multispectral imagery. The performance of various DL object-detection models was evaluated utilizing white pine training samples from the LD training site. The models included prominent architectures such as Faster R-CNN, SSD, RetinaNet, FoveaBox, FCOS, VFNet, Cascade R-CNN, and networks enhanced with PaFPN, and other models listed in Table 5. Model training incorporated rotated and non-rotated imagery to augment data diversity [54]. Model performance was assessed with average precision at an IoU threshold of 0.5 and high-confidence detection counts (≥95%).

Among all models, Faster R-CNN consistently achieved the highest average precision (Table 6, Figure 7) and detection count, particularly in RGB-rotated and multispectral-rotated scenarios, marking it the optimal architecture for further deployment. FoveaBox and PaFPN also performed competitively (average precision ~0.70–0.76), especially in non-rotated imagery. Empirical attention models and anchor-free detectors such as FCOS showed moderate average precision, while GHM and FSAF exhibited minimal detection of white pine, likely due to scale sensitivity or inadequate training convergence under limited sample conditions.

### 4.2. Model Optimization and Data Augmentation Effects

Data augmentation is a standard technique designed to enhance model robustness. However, our study found that its effectiveness depends on the specific method employed. We observed a counterintuitive result: disabling the default on-the-fly augmentation pipeline—which includes dynamic transformations such as zoom, crop, and brightness—improved performance significantly. For example, in the MD block, Faster R-CNN detected 2398 white pine crowns in the RGB-rotated configuration without augmentation, compared to only 942 with default augmentation. A similar trend was observed in the HD block for multispectral-rotated images (1471 vs. 944 detections).

This result highlights a key distinction in our methodology. We found that offline augmentation (creating a larger training set by rotating the source imagery before training) was beneficial, improving the model’s ability to recognize seedlings across multiple orientations. However, the additional, generalized on-the-fly augmentations hindered the final accuracy. This finding is consistent with research by [9], who also reported that an F-RCNN model did not always benefit from an on-the-fly augmentation pipeline. This suggests that, for our high-quality, consistent UAV imagery, the targeted application of rotation was the most valuable form of data diversity, whereas dynamic transformations may have introduced unrealistic visual artifacts that were counterproductive to the learning process.

### 4.3. Detection Performance by Crown Scale and Imagery Type

To ensure robust training, manually digitized white pine crowns ranging from 24 to 1276 pixels (mean: 297 pixels) were used to represent small to medium-scale crowns. We compared our results to those of Hao et al. [55], who identified an optimal “crown resolution” of 800–12,800 pixels as ideal for tree delineation using a Mask-RCNN model. While most of our samples fell below this range, our Faster R-CNN model demonstrated strong adaptability for the less complex task of tree detection at smaller object sizes. This difference in model objective (detection vs. delineation) and species characteristics (white pine vs. Chinese fir) likely explains why our effective detection range was smaller than the delineation-focused range identified by [54].

The performance of imagery varied with stand density. In the MD block, RGB imagery performed better, particularly when paired with model configurations such as the F-RCNN model. Conversely, in the more challenging HD block, multispectral imagery detected a greater number of trees. The limited advantage of multispectral data in the MD block is possibly because the key distinguishing features for these young pines were primarily structural and textural, which are well-captured by RGB imagery. The physiological information from the additional spectral bands may be less critical for detection at this early growth stage, as there is a potential misalignment between the available spectral features and the required regeneration traits.

### 4.4. Comparison with Classical Approaches

Classical approaches, such as LM and marker-controlled watershed segmentation (MCWS), often underperform in dense or overlapping canopy conditions [1]. As reported in [24], F1 scores of 87% and 91% were achieved for LM and 86% for MCWS, depending on window size and input type. Segmentation on stereo-derived digital elevation models (DEMs) showed poor performance when applied to complex canopies [3]. Similarly, the use of LM on UAV-CHM in open-canopy conifer forests achieved F1 scores above 0.80 but remained dependent on filter size and canopy openness [11]. DL approaches resolve many of these limitations. Studies by [18,21] demonstrated that CNN-based detectors, including F-RCNN, Mask-RCNN, and SSD, could achieve detection accuracies above 90% for loblolly pine and Chinese fir plantations. Yu et al. [24] further confirmed Mask R-CNN’s superiority over LM and MCWS by a 6–10% increase in F1 scores in young forest plantations. The findings corroborate the superiority of DL for detecting small and overlapping crowns.

### 4.5. Advances in Unsupervised and Hybrid Approaches

A primary limitation of the supervised DL approach used in this study is the labor-intensive nature of generating high-quality training data. The practical value of this methodology is therefore a matter of scale; for small sites, the significant upfront investment in manual data annotation may not be more efficient than a traditional field survey. However, for large-scale, operational inventories, this approach becomes highly valuable, as a well-trained model can analyze vast areas far more rapidly and consistently than ground crews.

To mitigate this, some recent studies have adopted hybrid or unsupervised pipelines. For instance, Jayathunga et al. [30] applied an unsupervised approach using UAV-digital aerial photogrammetry (DAP) point clouds, incorporating spatial, spectral, and structural features, achieving F1 scores of 96.6% for mapping regularly spaced conifer seedlings. The success of the method was partly dependent on the regular spacing of the planted seedlings, and such unsupervised approaches may struggle with species-specific detection or false positives in more complex, mixed-vegetation contexts where such clear spatial patterns are absent.

Hybrid approaches combining automated predictions with manual refinement or pre-trained weights offer a balanced compromise, supporting broader applicability while maintaining performance. For example, Weinstein et al. [23] used a semi-supervised approach, a LiDAR-based algorithm to generate over 400,000 unsupervised tree labels for initial model training, which was then improved with just under 3000 hand-annotated examples for individual tree crown detection. This demonstrates a massive reduction in the required manual effort. Weinstein et al. [56] demonstrated a transfer learning approach using a pre-trained model to achieve performance comparable to that of a model trained exclusively on local data, often requiring fewer than 1000 manually labeled tree crowns to reach near-optimal performance in tree crown detection. This task can be completed within a few hours.

### 4.6. Practical Considerations and Future Research

The use of ArcGIS Pro for end-to-end model training and detection offers significant advantages in usability and scalability, enabling forestry practitioners to deploy DL models without extensive programming expertise. This model-agnostic training interface facilitates flexible experimentation and standardization across forest types by integrating SSD, YOLOv3, RetinaNet, and Faster R-CNN.

However, several limitations remain. This study relied on imagery from a single November 2021 UAV flight, which limits analysis of phenological effects. The spatial resolution (6 cm) was constrained by flight height and canopy cover, restricting the detection of very small regeneration. Higher resolution, multi-temporal datasets and ground-truthing with high-accuracy GPS would significantly improve detection precision and transferability. In addition to these data-related constraints, UAV imagery is inherently subject to variability from illumination differences, shadowing, background clutter, motion blur, and sensor noise, which can influence object detection performance. In this study, robustness to such variability was partially addressed through data augmentation and by evaluating model transferability across regeneration density classes, which introduced natural variation in canopy structure and background complexity. However, under more extreme or unmodeled imaging conditions, detection performance may degrade, leading to increased FN in heavily shadowed areas or FP in complex backgrounds.

Beyond the fully supervised, axis-aligned detection approaches examined here, other object detection paradigms are increasingly being explored in remote sensing and may be relevant for future forestry applications. Weakly supervised object detection, for example, seeks to reduce the time and effort required for bounding-box annotation by relying on image-level labels during training. Recent work demonstrated that combining multiple-instance learning with complementary detection and instance difficulty evaluation can improve the detection of less salient objects in remote sensing imagery [57]. Adapting such approaches to forest regeneration mapping could help expand training datasets more efficiently, particularly across large or diverse landscapes.

In parallel, arbitrary-oriented object detection extends conventional detectors by predicting rotated bounding boxes that more tightly align with object geometry, thereby reducing background overlap and improving object separation in dense scenes. Attention-guided, orientation-aware models such as AG-Yolo have shown that incorporating rotation parameters and attention mechanisms can improve robustness in complex remote sensing imagery [58]. While these approaches were not evaluated in this study, which focused on operational workflows supported in ArcGIS Pro, they represent promising directions for improving detection robustness and precision in future forestry applications.

Additionally, this research addresses a key gap in the remote sensing field: the lack of standardized accuracy assessment protocols for DL-based object detection [59]. This study advances science by demonstrating a practical hybrid accuracy assessment strategy that integrates automated detection with manual mapping and validation.

In this workflow, the DL model detects the primary objects (white pine). Other land cover classes within the site are then manually delineated to complete the map. To assess this map’s accuracy, an independent, manually created reference dataset is used to build a complete confusion matrix. This allows for the calculation of robust, map-wide metrics (e.g., Overall, User’s, and Producer’s Accuracy) that account for both commission and omission errors across the entire landscape. While this hybrid assessment provides a robust solution, further refinement is needed to fully achieve probabilistic, population-level accuracy estimation for large-scale forest monitoring [59,60].

## 5. Conclusions

This study demonstrated the efficacy of DL-based object-detection models, notably Faster R-CNN, for detecting regenerating white pine using high-resolution UAV imagery. By systematically evaluating 20 architectures across RGB and multispectral imagery with rotated and non-rotated scenarios, we identified Faster R-CNN as the most robust and accurate. It achieved average precision values up to 0.88 and outperformed anchor-free and feature-enhanced alternatives such as FoveaBox and PaFPN.

Contrary to conventional expectations, models trained without default on-the-fly data augmentation but with data augmentation (image rotation in the training phase) consistently yielded superior detection and accuracy. This finding highlights the need for context-specific augmentation strategies, especially in structured forest environments where geometric transformations may introduce detrimental artifacts.

The study introduced a hybrid accuracy assessment framework that integrated standard DL evaluation metrics with traditional confusion matrix-based validation. While DL object-detection models are typically assessed using metrics like precision and recall (which primarily focus on detected objects and missed detections), these do not inherently account for TNs—areas where no objects exist and the model correctly makes no detection. Incorporating manually delineated reference polygons and “Other” class buffers allowed for the inclusion of TNs, enabling a more complete accuracy evaluation in line with best practices outlined in recent remote sensing literature [59]. This hybrid methodology provides a holistic understanding of model performance, bridging the gap between typical object detection metrics and the broader map-level accuracy required for remote sensing applications.

Overall, this research highlights the operational viability of ArcGIS Pro’s DL toolbox for white pine regeneration monitoring within the specific forest conditions of our study. It demonstrates that when paired with training data, appropriate model selection, and comprehensive validation, DL offers a powerful solution for enhancing forest regeneration assessments in similar contexts. Future work should explore model transferability across diverse tree species, long-term temporal monitoring of growth dynamics, and the integration of UAV-LiDAR for 3D crown structure refinement, thereby further clarifying the broader generalizability of these methods.

## Figures and Tables

**Table 1:** Plot-level summary statistics of white pine count, crown width, and height within the three stratified blocks.

Block	Variable	Mean	Median	St. Dev.	Min	Max
Low density	Count (stems/ha)	3657	9.00	3.36	4.00	12.00
	Crown Width (m)	1.08	0.94	0.59	0.08	2.83
	Height (m)	1.91	1.83	0.95	0.31	3.96
Medium density	Count (stems/ha)	4744	9.00	7.32	1.00	31.00
	Crown Width (m)	0.99	0.82	0.64	0.08	2.47
	Height (m)	1.28	1.22	0.68	0.31	3.36
High density	Count (stems/ha)	8796	4.00	6.16	1.00	25.00
	Crown Width (m)	1.03	0.96	0.58	0.09	2.77
	Height (m)	1.77	1.83	0.89	0.31	4.27

**Table 2:** Flight planning and imagery acquisition parameters.

Item	Value
Aircraft	DJI Matrice 100
Sensor	MicaSense RedEdge-M (RE)
Acquisition Date	8 November 2021
Flight Planning	Pix4Dcapture
Phenology	Leaf Off
Ground Sampling Distance (cm)	6
Flight Pattern	Single Grid
Side Overlap (%)	80
Front Overlap (%)	80
Flight Height (m)	93
Flight Speed (m/s)	8.9
Temperature (°C)	10
Cloud Coverage	Clear
Visibility (km)	16
GCPs	4

**Table 3:** UAV-derived products and image processing time.

Output Product	Processing Time
Initial Processing	15 min 45 s
Point Cloud Densification	25 min 42 s
Point Cloud Classification	4 min 34 s
3D Textured Mesh Generation	27 min 55 s
Digital Surface Model (DSM)	3 min 51 s
Orthomosaic	18 min 12 s
Digital Terrain Model (DTM)	55 s
Reflectance Map	31 min 14 s
Index Map	4 min 48 s

**Table 5:** The DL object-detection models examined in this study.

Model	Abbreviation	References
SingleShotDetector	SSD	[38]
RetinaNet		[34]
FasterRCNN	F-RCNN	[35]
YOLOv3	YOLOv3	[38]
Adaptive Training Sample Selection	ATSS	[52]
Content-Aware ReAssembly of FEatures	CARAFE	[47]
CascadeRCNN		[37]
CascadeRPN		[49]
Deformable Convolutional Networks	DCN	[46]
DynamicRCNN		[43]
EmpiricalAttention		[53]
Fully Convolutional One-Stage Object Detection	FCOS	[39]
FoveaBox		[40]
Feature Selective Anchor-Free Module	FSAF	[42]
Gradient Harmonizing Mechanism	GHM	[48]
LibraRCNN		[36]
Path Aggregation Network Feature Pyramid Network	PaFPN	[44]
Res2Net		[45]
Side-Aware Boundary Localization	SABL	[50]
VarifocalNet	VFNet	[41]

**Table 6:** The average precision score of the trained DL object-detection model trained with RGB and multispectral imagery, with and without image rotation.

Model	RGB		Multispectral	
	No Rotation	Rotation	No Rotation	Rotation
ATSS	0.54	0.48	0.47	0.53
CARAFE	0.49	0.66	0.48	0.63
Cascade RPN	0.00	0.27	0.34	0.43
CascadeRCNN	0.60	0.62	0.62	0.62
DCN	0.51	0.48	0.47	0.41
DynamicRCNN	0.31	0.21	0.51	0.41
Empirical Attention	0.43	0.67	0.42	0.58
FCOS	0.65	0.52	0.57	0.59
FoveaBox	0.73	0.71	0.70	0.60
F-RCNN	0.79	0.80	0.76	0.77
FSAF	0.00	0.68	0.65	0.52
GHM	0.14	0.10	0.58	0.15
LibraRCNN	0.35	0.68	0.31	0.65
PaFPN	0.69	0.66	0.59	0.66
Res2Net	0.62	0.65	0.57	0.72
RetinaNet	0.63	0.62	0.62	0.57
SABL	0.59	0.59	0.48	0.60
Singleshot	0.54	0.34	0.50	0.37
VFNet	0.66	0.67	0.58	0.60
YOLOv3	0.58	0.50	0.65	0.49

**Table 7:** Parameter values for training the DL object-detection model.

Parameter	Description	Assigned Values
Number of Epochs	The number of complete passes through the training dataset.	100
Batch Size	The number of images processed in each training step.	32
Validation %	Percentage of training data used for model validation.	10
Early Stopping	Stops training automatically if model performance stops improving.	Yes
Data Augmentation	Applying random transformation (Crop, brightness, contrast, zoom) to training images.	Default
Data Augmentation	No data augmentation.	No
Evaluation Metric	Weighted mean of precision at each threshold level.	Average Precision

**Table 8:** Parameter values used for red pine seedlings detection and delineation.

Parameter	Description	Assigned Values
Padding	The number of overlapping pixels applied to adjacent image tiles to ensure smooth and continuous predictions across tile boundaries.	56
Batch Size	The number of images processed in each training step.	32
Threshold	The final output results must include a minimum confidence score for a detection.	0.95
NMS	Process to eliminate redundant overlapping bounding boxes, retaining only the detection with the highest confidence for each object.	Checked

**Table 9:** Confusion matrix showing the classification performance of the FRCNN model on the MD test dataset. Rows represent reference polygon classes, and columns represent detection outcomes.

Class	Reference		
	Detected (White Pine)	Not Detected	Total
White Pine	412 (TP)	88 (FN)	500
Other	2 (FP)	498 (TN)	500
Total	414	586	1000

**Table 10:** Confusion matrix showing polygon-level classification performance of the FRCNN model on the HD test dataset. Rows represent reference polygon classes, and columns represent detection outcomes.

Class	Reference		
	Detected (White Pine)	Not Detected	Total
White Pine	434 (TP)	66 (FN)	500
Other	24 (FP)	376 (TN)	400
Total	458	442	900

## Data Availability

The data that support the findings of this study are available from the corresponding author upon reasonable request.
